# Transcriptome analysis of feather follicles reveals candidate genes and pathways associated with pheomelanin pigmentation in chickens

**DOI:** 10.1038/s41598-020-68931-1

**Published:** 2020-07-21

**Authors:** Xiaotong Zheng, Bo Zhang, Yawen Zhang, Haian Zhong, Ruixue Nie, Junying Li, Hao Zhang, Changxin Wu

**Affiliations:** National Engineering Laboratory for Animal Breeding, College of Animal Science and Technology, China Agricultural University, Beijing, 100193 China

**Keywords:** Genetics, Genetics, Gene expression, Gene expression

## Abstract

Yellow plumage is common in chickens, especially in breeds such as the Huiyang Bearded chicken, which is indigenous to China. We evaluated plumage colour distribution in F1, F2, and F3 populations of an Huiyang Bearded chicken × White Leghorn chicken cross, the heredity of the yellow plumage trait was distinguished from that of the gold plumage and other known plumage colours. Microscopic analysis of the feather follicles indicated that pheomelanin particles were formed in yellow but not in white feathers. To screen genes related to formation of the pheomelanin particles, we generated transcriptome data from yellow and white feather follicles from 7- and 11-week-old F3 chickens using RNA-seq. We identified 27 differentially expressed genes (DEGs) when comparing the yellow and white feather follicles. These DEGs were enriched in the Gene Ontology classes ‘melanosome’ and ‘melanosome organization’ related to the pigmentation process. Down-regulation of *TYRP1*, *DCT*, *PMEL*, *MLANA*, and *HPGDS*, verified using quantitative reverse transcription PCR, may lead to reduced eumelanin and increased pheomelanin synthesis in yellow plumage. Owing to the presence of the *Dominant white* locus, both white and yellow plumage lack eumelanin, and white feathers showed no pigments. Our results provide an understanding of yellow plumage formation in chickens.

## Introduction

Plumage colour in birds, coat colour in mammals, and skin colour in humans have long been the focus of pigment research. Among these, birds display the most fascinating and complex colouration, which has been shown to be caused by melanin (eumelanin and pheomelanin), carotenoids, porphyrins, polyenes and structural colours^1,2^. In general, the formation of different plumage colours in chickens is mainly attributed to variations in the quantity, proportion and location of eumelanin and pheomelanin in the feather^3^. Eumelanin makes plumage appear black and dark brown, whereas pheomelanin makes it appear red and yellow^4^. Both eumelanin and pheomelanin are indole-polymers with tyrosine as a precursor^5^. During eumelanin synthesis, tyrosinase (TYR) catalyses the hydroxylation of tyrosine to 3,4-dihydroxyphenylalanine (dopa) and the oxidation of dopa to dopaquinone, which is then oxidized to form eumelanin; this occurs via the TYR family, involving TYR, tyrosinase-related protein 1 (TYRP1), and dopachrome tautomerase (DCT). Dopaquinone is transformed into 5-*S*-cysteinyldopa when cysteine or glutathione provides sulfhydryl; it eventually forms pheomelanin^5^.

In recent decades, with the rapid development of sequencing technology, significant progress has been made in the study of functional genes related to plumage colour in chickens. Various plumage colour causal genes, such as the *Dominant white* gene (I), *recessive white* gene (c), *Silver* gene (S), *molted* gene (mo) and *Sex-linked barring* gene (B), have been cloned^6–11^. Especially for *Dominant white* allele (I), a strong dilutor of eumelanin owing to an insertion in the transmembrane region of the premelanosome protein (*PMEL*), it has no effect on the pigmentation of pheomelanin^6,12,13^. Therefore, it is widely used in the breeding of white-feathered chickens. In addition, many genes, such as those in the tyrosinase (*TYR*) gene family, as well as melanocortin 1 receptor (*MC1R*) and melanogenesis associated transcription factor (*MITF*), have been found to play important roles in melanin synthesis^6,14,15^. Mutations in the *PMEL*, solute carrier family 45 member 2 (*SLC45A2*), and cyclin-dependent kinase inhibitor 2A (*CDKN2A*) can alter melanin content, generating specific plumage colours^6,9,11^. The formation of plumage colour is also influenced by the expression of various genes related to pigment formation in feather follicles during the growth period^16^. As the pivotal position for feather formation and melanogenesis, transcriptome profiling of the feather follicle has been performed to elucidate the process of morphogenesis and to identify candidate genes of diverse feather shapes and colours^17–23^.

**Figure 1:**
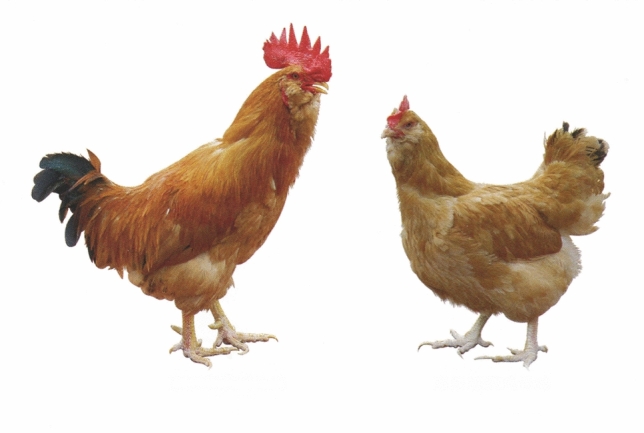
The exterior and plumage colour of Huiyang Bearded chicken^29^. This image cited from the Animal Genetic Resources in China Indigenous Breeds provided by China National Commission of Animal Genetic Resources.

Yellow plumage is common in Chinese indigenous chicken populations. It is considered a marker of high-quality meat-type chickens, represented by Huiyang Bearded chickens (HB), which are very popular in the Chinese consumer market. The plumage colour (Fig. 1) is light yellow and is distinct from that of the commercial strain (Rhode Island Red chicken) with gold plumage by visual observation^24,25^. A causal mutation responsible for a similar yellow plumage phenotype (an 8.3 kb deletion upstream of the *SOX10* gene) has been reported in chickens^26^. Polymorphisms in *MC1R* are related to plumage colour variations in Golden duckwing Araucana chickens^27^. A non-synonymous T-to-C substitution in *TYRP1* is a causal mutation leading to a roux mutation in Japanese quail^28^. However, none of the above mutations were found in the HB population, which suggests that the formation mechanisms of yellow plumage of Chinese native chicken was different from the previously studies.

In this study, a hybrid population of HB and White Leghorn chickens (WL) was established to observe the inheritance of yellow plumage. In the offspring population, the *Dominant white* allele was found to have no epistatic effect on yellow pluamge, which is different from brown, red, and other known plumage colours. Therefore, we collected the feather follicles of yellow and white plumage chickens (both with the *Dominant white* allele), and performed dynamic transcriptome profiling to identify candidate genes for yellow plumage in chickens. This study provides a foundation for plumage colour selection in chicken breeding.

## Results

### Plumage colour distribution in experimental chickens

The Huiyang Bearded chicken, which is native to the Guangdong province, China, has yellow plumage in both sexes (Fig. 1)^29^. The White Leghorn chicken, which is homozygous for the *Dominant white* gene, has white plumage. In the F1 population (684 cocks and 685 hens) bred from crossing male HB with female WL chickens, all cocks had white plumage; among the hens, plumage colours were distributed as follows: 5% white, 5% grey, 45% yellow, and 45% grey plume yellow head. The F2 population had the following plumage colour distribution in both cocks and hens: white (49%), yellow (28%), grey (12%), black (1%) and black and white barred (10%) (Supplementary Figure S1, Supplementary Table S1). Only the yellow plumage breeders of F2 were selected to reproduce an F3 population, of which 37% had white plumage.

### Genetic analysis of the yellow plumage trait

All F0 HB cocks had yellow plumage, with the *Silver* locus (ss) and *Dominant white* locus (ii) genotypes, whereas all F0 WL hens were white, because they had the *Dominant white* locus (II) and *Silver* locus (S/W) genotypes. In the F1 hens, the *Dominant white* locus had the Ii genotype, and the *Silver* locus had the s/W genotype. However, a high proportion of yellow plumage individuals was observed among the F1 hens, which indicates that the gene controlling yellow plumage had an epistatic effect on the *Dominant white* gene. The presence of multiple plumage colours in F1 hens also suggests that the yellow plumage gene may be influenced by other plumage colour genes. In F1 cocks, the *Dominant white* locus had the Ii genotype and the *Silver* locus had the Ss genotype; the fact that all of the F1 cocks had white plumage indicates that the *Silver* gene had epistatic effects on the yellow plumage gene (Supplementary Figure S2). In both F1 and F2 individuals with white plumage, the frequency of the *recessive white* locus (cc) was zero, indicating that their white plumage was not caused by the *recessive white* allele (c). The emergence of black and white barred individuals in the F2 population was verified to be caused by the *Sex-linked barring* (B) allele. Interestingly, we found white barring on a yellow background in a few light yellow plumage chickens, similar to the black and white barred pattern; the presence of the B allele was also identified in these individuals. This shows that a yellow and white barred pattern plumage can occur when both B and yellow plumage alleles exist. In summary, these results suggest that the yellow plumage gene had an epistatic effect on the *Dominant white* gene, whereas the yellow plumage gene was hypostatic to the *Silver* gene; further, they suggest that the effects of the yellow plumage gene and *Sex-linked barring* gene are independent of one another.

We then identified the *Dominant white* and *Silver* loci in yellow and white plumage individuals in the F3 population. Six yellow and six white plumage individuals were sampled for RNA-seq analysis. They were homozygous in the *recessive white* locus (CC) and *Silver* locus (s/W), and homozygous (II) or heterozygous (Ii) in the *Dominant white* locus (Supplementary Table S2). This indicates that the phenotypic differences between the yellow and white plumage chickens were not due to these gene loci.

### Pigment deposition via histological analysis of feather follicles

**Figure 2:**
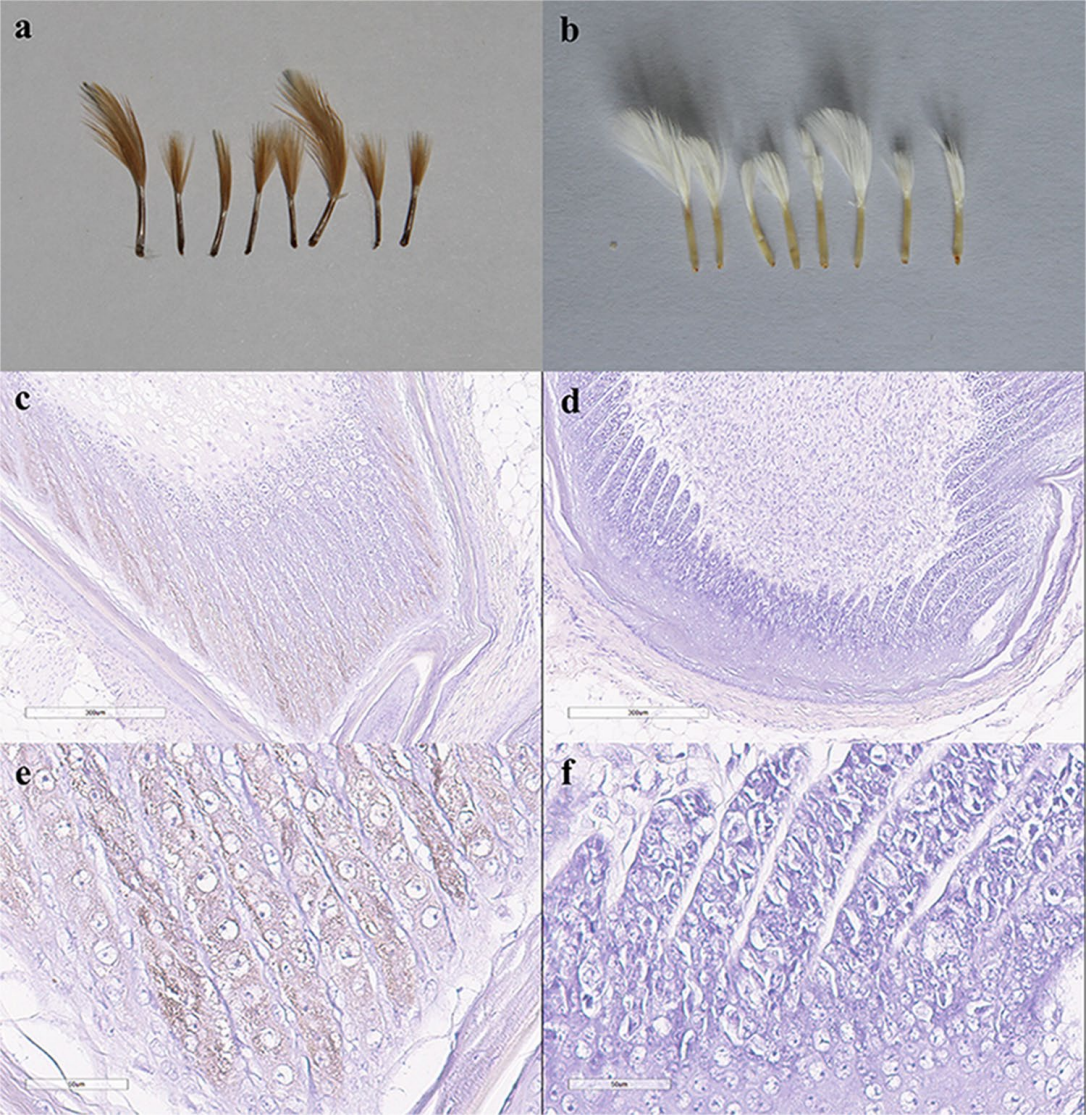
Characterisation of yellow and white phenotypes. (**a**) Feather follicles from yellow plumage types. (**b**) Feather follicles from white plumage types. (**c**–**f**) Haematoxylin-stained sections of newly emerged feather follicles from yellow (**c**, **e**) and white (**d**, **f**) chicken. Magnified area in (**c**, **d**) are shown in (**e**, **f**), respectively. Scale bar in (**c**, **d**) 300 μm. Scale bar in (**e**, **f**) 50 μm.

Histological sections of newly emerged yellow and white feather follicles revealed that they had similar cellular composition, with abundant keratinocytes and dermal cells. However, yellowish-brown particles were present in the yellow feather follicles, but not in white feather follicles (Fig. 2). As pheomelanin is the only type of melanin that can make a feather appear yellow, our preliminary conclusion was that yellow plumage colour was caused by increased pheomelanin production. As expected, eumelanin was not observed in the feather follicles of either plumage colour, because of the presence of the *Dominant white* locus (I).

### Summary of RNA sequencing data

Twelve libraries were sequenced from colourless RNA solutions obtained from samples of 12 individuals (white plumage from three 7-week-old chickens: 7W1, 7W2, and 7W3; yellow plumage from three 7-week-old chickens: 7Y1, 7Y3, and 7Y4; white plumage from three 11-week-old chickens: 1W1, 1W3, and 1W4; and yellow plumage from three 11-week-old chickens: 1Y1, 1Y3, and 1Y4); 140.7 Gb of data was obtained. The mapping rate of the filtered sequencing data to the reference genome exceeded 80%, and the GC content per library was more than 50% (Supplementary Table S3). For the uniquely aligned sequences in the gene annotation files, the numbers and proportions of three functional elements (mRNA, introns and intergenic regions) were obtained: 76.37% ± 2.46% reads per sample were aligned to the mRNA region (Supplementary Figure S3).

In total, 10,036–10,070 expressed genes were detected. On average, 52.68% of the expressed genes had low expression levels (fragments per kilobase million mapped (FPKM) < 1) in the feather follicles. A few genes had extremely high expression levels (FPKM ≥ 500); these accounted for 1.65% of the total number (Supplementary Figure S4).

### Differentially expressed genes between the yellow and white feather follicles

**Figure 3:**
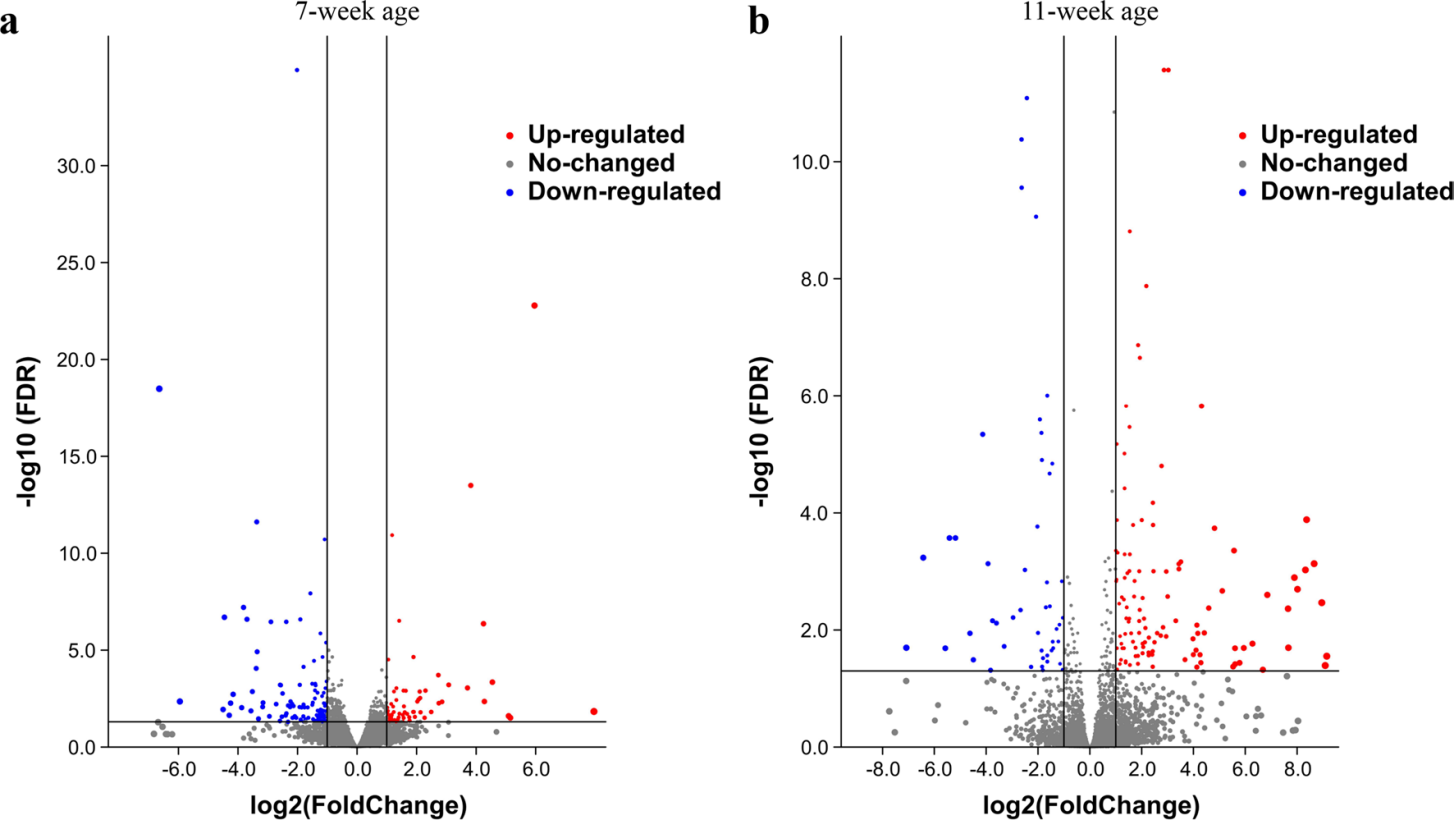
Volcano plot of differentially expressed genes (DEGs) in RNA-seq. (**a**) Volcano plot of differentially expressed genes (DEGs) in RNA-seq of 7-week age. (**b**) Volcano plot of differentially expressed genes (DEGs) in RNA-seq of 11-week age.

Comparing the gene expression levels between yellow and white feather follicles, 177 differentially expressed genes (DEGs) were obtained in the 7-week age class and 163 DEGs in the 11-week age class (Fig. 3, Supplementary Table S4). Gene Ontology (GO) pathway enrichment analysis revealed the DEGs in the 7-week age class were mainly enriched in the GO classes ‘melanosome’, ‘lysosomal membrane’, and ‘early endosome’. Kyoto Encyclopedia of Genes and Genomes (KEGG) pathway enrichment analysis for the 7-week age class found that ‘melanogenesis’ and ‘metabolic pathways’ were enriched. The DEGs in the 11-week age class were mainly enriched in the GO classes ‘melanosome’, ‘melanosome organization’, and ‘integral component of plasma membrane’(Table 1).

**Table 1:** Results of GO and KEGG enrichment analysis of differentially expressed genes (DEGs) using the DAVID database.

Group	Category	Term	*P*-value	Genes
DEGs of 7-week age	GOTERM_CC_DIRECT	Melanosome	4.23E−05	*DCT, TYRP1, PMEL, MLANA*
	GOTERM_CC_DIRECT	Lysosomal membrane	0.0078	*ATP11C, ENPEP, MFSD12, ATP6V0D2, DPP4*
	KEGG_PATHWAY	Melanogenesis	0.0086	*DCT, WNT16, TYRP1, POMC*
	GOTERM_CC_DIRECT	Early endosome	0.0332	*CLCN3, TRAK1, NEURL1B, ATP6V0D2*
	GOTERM_CC_DIRECT	Cytoplasm	0.0359	*GNA13, CTHRC1, IL1R2, EPAS1, AKAP12, ENPEP, VASH2, PADI1, NECAB1, DPF1, BRINP1, DIP2B, MAP3K1, RGS6, SERPINB10, SMURF1, ITCH, YAP1, FBXL15, HPGDS, WWOX*
	GOTERM_MF_DIRECT	Ligase activity	0.0420	*NEURL1B, SMURF1, ITCH*
	GOTERM_BP_DIRECT	Glomerulus development	0.0439	*PLCE1, ENPEP*
	KEGG_PATHWAY	Metabolic pathways	0.0495	*DCT, MINPP1, PLCE1, TYRP1, NANS, HMGCS2, FBP1, ATP6V0D2, GLCE, HPGDS*
DEGs of 11-week age	GOTERM_CC_DIRECT	Melanosome	4.23E−05	*RAB32, TYRP1, PMEL, SYTL2, MLANA*
	GOTERM_BP_DIRECT	Melanosome organization	0.0020	*RAB32, TYRP1, PMEL*
	GOTERM_CC_DIRECT	Integral component of plasma membrane	0.0043	*OPN5L2, KCNJ16, STEAP4, FOLH1, TSPAN10, AQP9, PMEL, AQP4, ADRA2C, TM4SF1, DSCAM*
	GOTERM_BP_DIRECT	Positive regulation of cell-substrate adhesion	0.0065	*EDIL3, COL8A1, VIT*
	GOTERM_BP_DIRECT	C-terminal protein deglutamylation	0.0202	*FOLH1, AGBL1*
	GOTERM_BP_DIRECT	Cell–cell signaling	0.0299	*LVRN, ADRA2C, POMC*
	GOTERM_MF_DIRECT	Hydrogen-exporting ATPase activity, phosphorylative mechanism	0.0324	*ATP6V1G3, ATP6V0D2*
	GOTERM_CC_DIRECT	Vacuolar proton-transporting V-type ATPase complex	0.0405	*ATP6V1G3, ATP6V0D2*
Overlapping DEGs	GOTERM_CC_DIRECT	Melanosome	4.35E−04	*TYRP1, PMEL, MLANA*
	GOTERM_BP_DIRECT	Melanosome organization	0.0117	*TYRP1, PMEL*

*CC* cellular component, *MF* molecular function, *BP* biological process.

There were 27 overlapping DEGs that occurred in both age groups (Supplementary Figure S5, Supplementary Table S4). These were significantly enriched in the two pigment-related GO classes ‘melanosome’ and ‘melanosome organization’. The genes involved included *PMEL*, *TYRP1* and *MLANA* (Table 1). Another overlapping DEG, *HPGDS*, which is involved in catalysing conjugation reactions with glutathione, might play an important role in pheomelanin synthesis.

### Expression profiles of key pigment genes and verification of RNA-seq data

**Figure 4:**
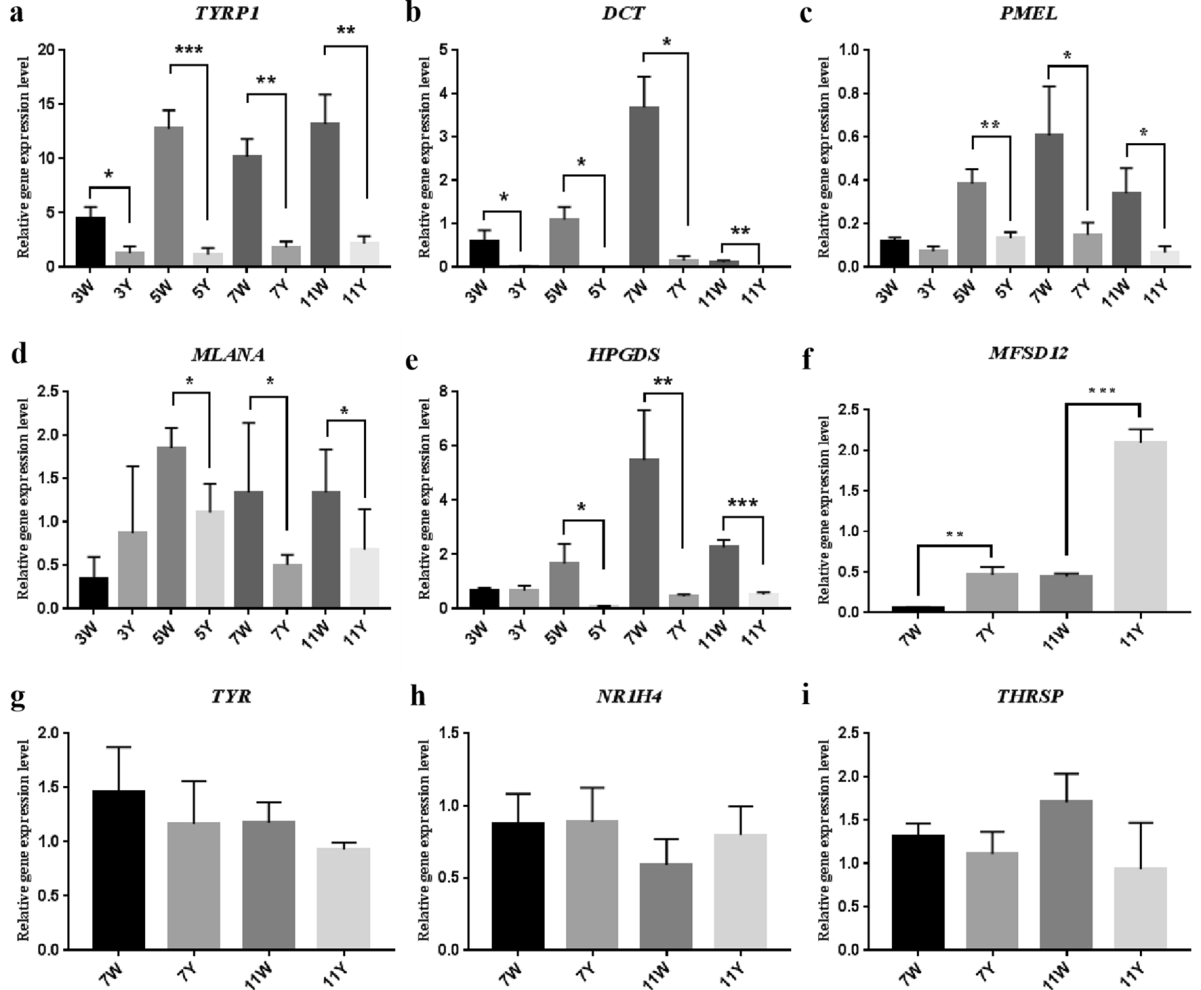
Verification of gene expression level via quantitative real-time polymerase chain reaction (qRT-PCR). *TYRP1*, *DCT*, *PMEL*, *MLANA*, and *HPGDS* expression (**a**–**e**) comparisons between white and yellow feather follicles in 3- (3W and 3Y), 5- (5W and 5Y), 7- (7W and 7Y), and 11-week (11W and 11Y) ages. *MFSD12*, *TYR*, *NR1H4*, and *THRSP* expression (**f**–**i**) comparisons between white and yellow feather follicles in 7- (7W and 7Y) and 11-week (11W and 11Y) ages. Sample size in each group was five. **Indicates extremely significant difference (P < 0.01), *indicates significant difference (P < 0.05).

The expression levels of five key pigment genes (*TYRP1*, *DCT*, *PMEL*, *MLANA* and *HPGDS*) were measured using real-time quantitative reverse transcription PCR (qRT-PCR) of samples from feather follicles at various time points (3, 5, 7, and 11 weeks of age). The results showed that the expression of *TYRP1* and *DCT* was lower in the yellow plumage group than in the white plumage group at the four ages, and the differences were significant (*P* < 0.05). Expression of *PMEL*, *MLANA,* and *HPGDS* genes was significantly lower in the yellow feather follicles than in the white feather follicles in chickens of 5, 7 and 11 weeks of age (*P* < 0.05); however, the differences were not significant in the 3-week age class (Fig. 4). This may be owing to the fact that at the 3 weeks of age, chicks are not yet at a critical time to moult. Among these five genes, *TYRP1* and *DCT* expression levels were approximately tenfold lower in yellow than in white feather samples in 5-, 7- and 11-week old chickens, suggesting that these two genes might participate in the pigmentation and control of plumage colour in chickens.

In addition to the five key pigment genes described above, we selected one DEG (*MFSD12*) and three non-DEGs (*TYR, NR1H4* and *THRSP*), and used qRT-PCR to measure gene expression in the feather follicles of 7- and 11-week old chickens, to verify the RNA-seq analysis results. *MFSD12* showed significant differences in the expression levels (*P* < 0.05), whereas the three non-DEGs showed no significant differences (Fig. 4). This indicates that the RNA-seq analysis results are reliable.

## Discussion

In birds, plumage colour variations are due to alterations in melanin production, distribution or deposition in feathers, and feather follicles are the only place where plumage melanin is produced^30^. In feather follicles, mature melanocytes produce melanosomes for melanogenesis; the newly synthesized melanin is then transferred to the keratinocytes of feathers, giving the feathers their particular colour^31^. There are two types of melanosomes, eumelanosomes and pheomelanosomes. Eumelanosomes are elliptical entity with a fibrillar matrix, and are responsible for eumelanin synthesis. Pheomelanosomes vary in shape, being predominantly rounded, and are responsible for the pheomelanin synthesis^32,33^. Our paraffin sections of feather follicles were not sufficiently magnified to observe melanosome structure and enable determination of the type. Nonetheless, we could identify the type of melanin produced by the colour displayed, and concluded that the pheomelanin, but not eumelanin, production was higher in the yellow than in the white feather follicles. Yellow or red coat colour has been shown to be associated with high levels of pheomelanin production: a 100-fold increase in the pheomelanin content was observed in the dorsal hairs of recessive yellow mice compared to black mice^34^. Higher levels of pheomelanin markers TTCA and 4-AHP were also observed in recessive red pigeons than in wild-type pigeons, based on the histology of regenerating feathers and chemical analysis of feather melanin^17^. Five down-regulated DEGs (*TYRP1*, *PMEL*, *MLANA*, *HPGDS* and *TSPAN10*) in our yellow plumage chickens were shared with the identified DEGs in recessive red pigeons^17^. As described in their hypothesis, pheomelanin-based phenotypes may result from changes in multiple pigmentation genes, reducing overall pigmentation and converting melanogenesis to pheomelanin production^17^. Our results are therefore consistent with previous studies, and confirmed that high levels of pheomelanin production contributed to the yellow plumage trait in our study.

In this study, we found that the *Dominant white* locus did not inhibit the pheomelanin synthesis and deposition. The *Dominant white* allele can alter the PMEL amyloid formation from functional to pathogenic, and inhibit eumelanin synthesis^35,36^. Similarly, previous studies have revealed that functional mutations in *PMEL* do not affect the production of pheomelanin in silver coat horses, silver mutation mouse, merle pattern domestic dogs, and yellowish plumage Japanese quail^12,13,37,38^. These results are understandable, given that the transmembrane glycoprotein encoded by *PMEL* is the main component of the fibrillar matrix of eumelanosomes rather than a component of pheomelanosomes^33,39^. A PMEL-silencing assay revealed that the mRNA expression of melanin biosynthesis genes, including *TYR*, did not differ from that in the control group^40^. This suggests that the *Dominant white* allele was functional at the protein level, but had no effect on the mRNA expression of melanin biosynthesis genes in our study. Some missense mutations of *SLC45A2* resulted in a dominant *Silver* locus that made yellow plumage undetectable, confirming the previous reported dilution effect on pheomelanin by this locus^9^. Similarly, dilution of pheomelanin with no effect on eumelanin, or with only a slight effect, has been observed in *SLC45A2* mutation horses^41^. It is interesting feature also an enigma concerning the specific inhibition of pheomelanin in *Silver* chickens. When the *Sex-linked barring* locus was present, the feathers of yellow plumage chickens showed a yellow and white barred pattern. This pattern can be caused by the B allele, the causative mutations are four single-nucleotide polymorphisms located in the *CDKN2A* gene^11^. These mutations cause high expression of *CDKN2A* and lead to the premature differentiation of melanocyte progenitor cells and premature depletion of melanocytes, this causes a white bar to appear where the melanocytes are absent^11^. Similarly, the diverse of feather patterns in pigeons are controlled by the distribution of melanocytes^16^. Lin et al.^42^ systematically explored the cellular and molecular basis of avian pigment pattern formation; they concluded that the diversity of avian pigment patterns is the result of modulating the presence, arrangement, or differentiation of melanocytes. Our findings elucidate the effect of several known pigment genes on yellow plumage; this may be helpful for the breeding of yellow plumage chickens.

**Figure 5:**
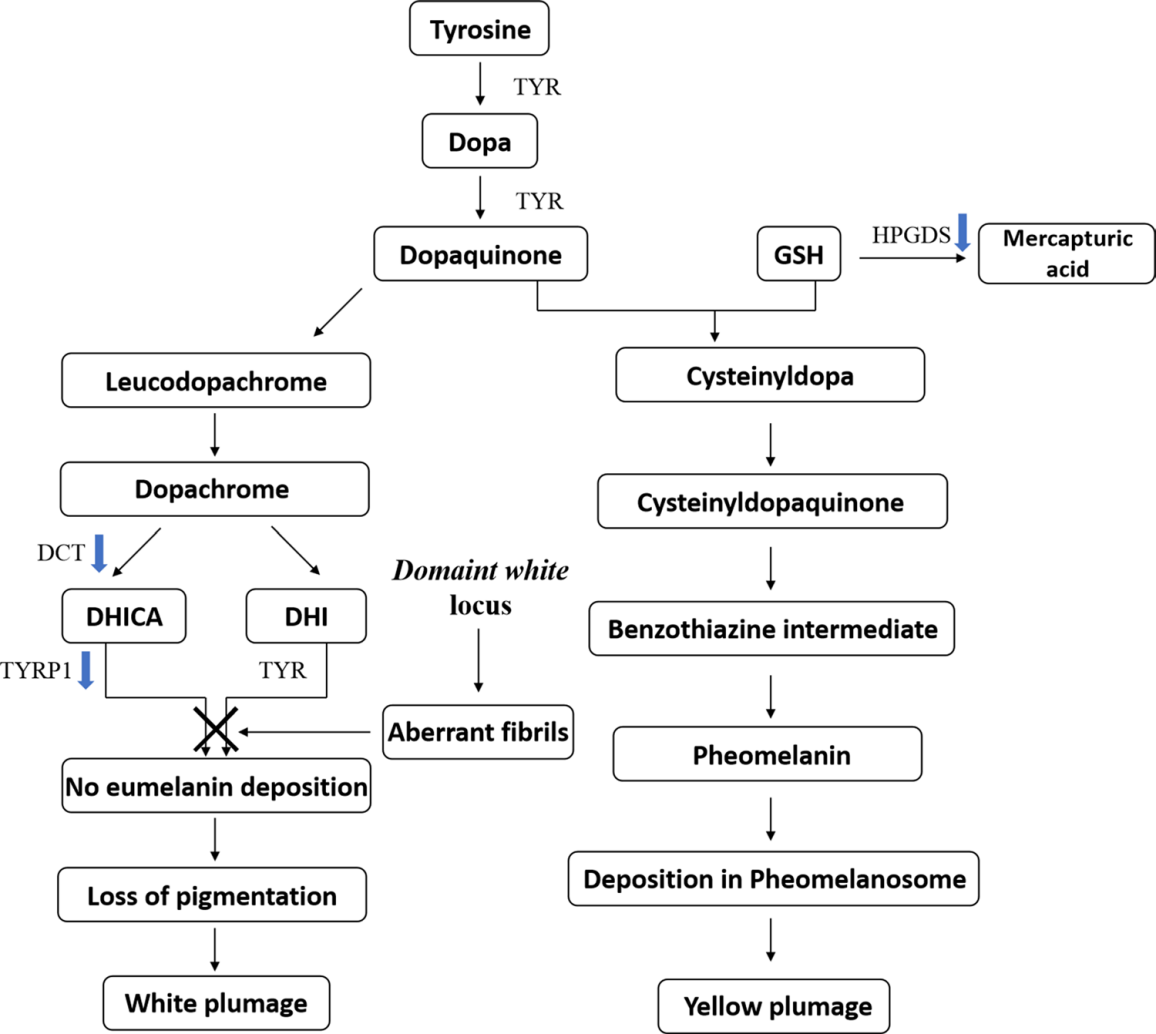
The synthetic process of eumelanin and pheomelanin. The blue arrow means that its corresponding coding gene is down-regulated in the yellow samples relative to the white samples. *Dopa* 3,4-dihydroxyphenylalanine, *DHICA* 5,6-dihydroxyindole-2-carboxylic acid, *DHI* 5,6-dihydroxyindole, *GSH* glutathione, *TYR* tyrosinase, *TYRP1* tyrosinase-related protein 1, *DCT* dopachrome tautomerase, *HPGDS* haematopoietic prostaglandin D2 synthase.

Melanin production is determined by relevant enzymes and their metabolic capacity. For example, the presence of TYR allows the rapid oxidation of tyrosine to dopaquinone, thus initiating the eumelanin or pheomelanin synthesis pathways in melanocytes^43^. Mutations in the *TYR* gene can lead to severe inadequacy in melanin production. The recessive white feather colour in chickens is caused by the insertion of a retroviral sequence on intron 4 of the *TYR* gene, resulting in a substantial reduction in the expression of normal transcript that contains exon 5^8^. The amount or activity of TYR, TYRP1, and DCT is also directly related to eumelanin content, because of their roles in the final step of eumelanin synthesis^43^. Further, the type of melanin produced is directly determined by the amounts of sulfhydryl compounds such as cysteine and glutathione that are present in the tissue (Fig. 5)^43,44^. Thus, the presence and activity of enzymes that catalyse glutathione reactions, such as glutathione peroxidase, glutathione reductase and glutathione *S*-transferase, will modify the progression of melanogenesis^45^. In previous studies, omics data were used to explore feather colour formation-related genes. The formation of Columbian plumage in chickens was related to changes in the expression of *MED23*, *FZD10*, *WNT7B* and *WNT11*^21^. DEGs between black and white feather colours in ducks were enriched in the pathways of melanogenesis (*c-Ki*t/*TYR*/ *TYRP1*) and tyrosine metabolism (*TYR*/ *TYRP1*)^23^. In our study, DEGs were significantly enriched in multiple pathways related to melanin synthesis, and were concentrated in several genes. Down-regulation of five pigment-related genes (*TYRP1*, *DCT*, *HPGDS*, *PMEL* and *MLANA*) was confirmed by qRT-PCR in 5-, 7- and 11-week old chickens. The enzymes or proteins encoded by these five genes can participate in melanogenesis by acting as catalysts or providing sites for melanin synthesis^46–50^. Therefore, it is possible that functional changes in enzymes or precursors, caused by the down-regulation of these five pigment-related genes, lead to an increase in pheomelanin synthesis.

*TYRP1*, the first pigment gene cloned in the tyrosinase gene family, encodes tyrosinase-related protein 1, a putative membrane-bound, copper-containing enzyme that plays a key role in the last step of eumelanin synthesis^46,51^. Previous studies have demonstrated strong correlations between changes in TYRP1 function and the pheomelanin-based coat colour of Japanese quail^28^, chickens^52^, cats^46^, rabbits^53^, and cattle^54^. Low expression levels of *TYRP1* were associated with a pheomelanin-based coat colour. In French cattle breeds, the greatest expression of *TYRP1* occurred in the black skin of Prim’Holstein cattle; in the pheomelanin-rich coats of the Blonde d’Aquitaine (blond), Limousin (red), and Salers (reddish brown) breeds, there were varying levels of complete repression of *TYRP1*^55^. In catfish, lower expression levels of *TYRP1* mRNA were detected in the albino yellow group than in the black wild-type group^56^. These results provide useful data for understanding the relationship between the lower expression of *TYRP1* and the pheomelanin-based phenotype. DCT is an enzyme that metabolises dopachrome (DC) to 5, 6-dihydroxyindole-2-carboxylic acid (DHICA) during eumelanin synthesis^47^. The slaty mutation is known to reduce the activity of DCT. In cultured slaty melanocytes and slaty mutation mice, the content of eumelanin was reduced, whereas the content of pheomelanin was greatly increased^57,58^. DCT knockout mice showed a diluted coat colour relative to the non-knockout mice^59^. These studies indicate that *DCT* repression can affect both eumelanin and pheomelanin synthesis. Additionally, we confirmed that there was no significant difference in *TYR* expression between the yellow and white feather follicles. Thus, reduced *TYRP1* and *DCT* levels caused a switch from eumelanin synthesis to pheomelanin synthesis (Fig. 5), which is a general trend under suppression of eumelanin synthesis^34^.

Haematopoietic prostaglandin D2 synthase (HPGDS) is a member of the *sigma*-class glutathione *S*-transferases (GSTs)^60^. HPGDS can be utilised as a cofactor in a variety of detoxification reactions, by catalysing the conjugation of mutagens and carcinogens with glutathione during mercapturic acid synthesis^49,61^. As a nucleophile, glutathione is the most abundant sulfhydryl compound in animal tissues and occurs mainly in the cytoplasm of aerobic organisms^62^. Glutathione levels are closely associated with the deposition of melanin in the coat or skin of vertebrates^63^. High concentrations of glutathione allow it to bind with dopaquinone to form 5-*S*-cysteinyldopa, thereby initiating the pheomelanin synthesis pathway. When the glutathione concentration decreases, dopaquinone is converted directly to dopachrome and ultimately forms eumelanin^38^. In melanoma cells, low levels of glutathione have been found to be associated with eumelanin-type pigmentation, while high levels were associated with pheomelanin-type pigmentation^64,65^. In mice, yellow hair follicles have high glutathione and total thiol concentrations, while black hair follicles have low glutathione concentrations^66^. Thus, we inferred that a reduction in *HPGDS* expression caused reduced mercapturic acid synthesis, which further increased pheomelanin synthesis in the yellow feather follicles. Our finding indicates that reduction in HPGDS might result in more glutathione providing sulfhydryl to participate in pheomelanin synthesis (Fig. 5).

PMEL is a melanosome matrix protein responsible for the formation of PMEL fibrils within eumelanosomes. PMEL fibrils are essential for the optimisation of normal eumelanosome morphogenesis and eumelanin deposition, as animals lacking *PMEL* expression exhibit varying degrees of eumelanin reduction^6,12,39,48,67^. Reduced expression of *PMEL* was found in Japanese quail with the yellowish phenotype^13^. Therefore, the loss of PMEL may also be one of the reasons for the formation of yellow plumage. *MLANA*, which encodes a melanoma antigen (melan-A) that is recognised by cytotoxic T lymphocytes, has been cloned while searching for melanoma antigens; it has been widely studied as a melanoma-specific antigen and melanosome-specific marker^68^. The expression of *MLANA* is observed only in melanocytes, melanomas, and retinal pigment epithelium, suggesting its association with melanogenesis^68,69^. Melan-A can form complexes with PMEL17, affecting both its stability and the processing required for melanosome structure and maturation, and participating in eumelanin synthesis^50^. In mice, knocking out the *MLANA* gene results in a reduction in melanin content. Likewise, the melanosomes of melanocytes in *MLANA* knockout mice showed morphological abnormalities, indicating that *MLANA* acts as a pigment gene for the biogenesis and maintenance of melanosomes^70^. Considering the above results, we believe that reduced expression of *PMEL* and *MLANA* also causes reduced eumelanin synthesis.

For yellow and white plumage formation, we inferred that the five pigment-related genes discussed cause a reduction in eumelanin and an increase in pheomelanin synthesis in yellow plumage. Further, due to the presence of *Dominant white* locus, both white plumage and yellow plumage lack the eumelanosomes required for normal eumelanin synthesis. Finally, only pheomelanin was detected in the yellow plumage samples, and none of two types of melanin was detected in the white plumage samples.

In conclusion, by analysing the plumage colour distribution among the offspring populations of HB × WL chickens, we revealed the heredity of the yellow plumage gene. The yellow plumage of HB was due to increased pheomelanin synthesis in the pheomelanosomes in the feather follicles. The transcriptome profiles and qRT-PCR validation revealed that down-regulation of *TYRP1*, *DCT*, *PMEL*, *MLANA* and *HPGDS* may alter eumelanin and pheomelanin content, leading to the formation of yellow plumage. We speculate that the phenotypic characteristics of the yellow plumage colour might be caused by the reduced expression of these genes. These findings provide new insights for future research in yellow plumage chickens.

## Methods

### Experimental animals and sampling

The F1 population was obtained by crossing HB cocks with WL hens. Yellow plumage females and white plumage males were selected from the F1 population to produce an F2 population. Yellow plumage females and males were then selected from the F2 population to produce an F3 population.

For the transcriptome sequencing and to verify gene expression, five white plumage and five yellow plumage chickens were randomly collected from the F3 population, and several newly emerged feathers were plucked from nape of the neck at four time points (3, 5, 7 and 11 weeks of age). The feather follicles were cut off 2 mm from the bottom end of the feather and then immediately put into RNA storage solution and stored at − 80 °C. For histological measurements, yellow and white plumage chickens at 11 weeks of age were bled to death, and the newly emerged feather follicles, along with the nearby skin, were removed and fixed in 4% paraformaldehyde at 4 °C. Animal treatment protocols were approved by The Beijing Municipal Committee of Animal Management and comply with guidelines of The Ethics Committee of the China Agricultural University.

### Identification of known genes associated with plumage pigment

In all of the transcriptome samples, three loci that cause white plumage were identified; these are the *Dominant white* locus on the *PMEL* gene; the sex-linked *Silver* locus, caused by a mutation of *SLC45A2* on the Z chromosome; and the *recessive white* locus, which results from the insertion of a complete avian retroviral sequence of 7.7 kb in intron 4 of the *TYR* gene^6–9^. Blood samples were collected from the brachial veins of chickens using standard venepuncture. Genomic DNA was extracted using the phenol and chloroform method^72^. The *Dominant white* and *recessive white* loci were identified using patented methods. PCR of the target region and Sanger sequencing were used to verify the base mutation of *SLC45A2*. Prior to this, the identification of the three loci was carried out in individuals of the F0, F1, and F2 populations, to elucidate the heredity of the yellow plumage trait.

### Histology of the feather follicles

The feather follicles, fixed at 4 °C overnight, were dehydrated in ethanol, cleaned using xylene, and embedded in paraffin. Longitudinal sections of 7 μm thickness were sliced and stained with haematoxylin using standard protocols; eosin was not used because it interferes with the visualisation of melanin. After haematoxylin staining, the sections were fixed with neutral resin and observed under a microscope.

### RNA extraction, library construction, and sequencing

Three white and three yellow feather follicle samples from chickens aged 7 and 11 weeks were selected for transcriptome sequencing. Total RNA was extracted from the feather follicles, including epithelium and pulp, using an RNAprep Pure Tissue Kit (Tiangen, Dalian, China) following the manufacturer’s instructions. RNA purity was checked using the NanoDrop 2000 spectrophotometer, and the concentration and integrity of RNA were assessed using the Agilent 2100 Bioanalyzer and Agilent RNA 6000 Nano Kit, respectively. In total, 2 μg RNA per sample was used as input material for RNA sample preparation. The sequencing libraries were prepared using the NEBNext^®^ Ultra™ RNA Library Prep Kit for Illumina^®^ (#E7530L, NEB, USA) following the manufacturer’s recommendations; the libraries were sequenced on an Illumina Hiseq 4000 platform using 150 bp paired-end reads. All RNA sequence data were deposited in the NCBI Gene Expression Omnibus under Accession GSE146956.

### Analysis of RNA-seq data

A Perl script was used to filter the original raw data. Contaminated adapter reads, reads with > 5% ambiguous “N” bases, and low quality reads were removed. The reference genomes (ftp://ftp.ensembl.org/pub/release-96/fasta/gallus_gallus/dna/Gallus_gallus.GRCg6a.dna.toplevel.fa) and the annotation file (ftp://ftp.ensembl.org/pub/release-96/gtf/gallus_gallus/Gallus_gallus.GRCg6a.96.gtf.gz) were downloaded from the ENSEMBL database (https://www.ensembl.org/index.html). The reference genome index was built using Bowtie2 v2.2.3, and the clean data were then aligned to the reference genome using TopHat2^72^. Reads for each gene were counted using HTSeq v0.6.0; the expression levels of genes per sample, expressed as FPKM, were based on sequence lengths and on the read counts mapped to them^71^.

### Screening and functional annotation of DEGs

Differential expression analysis between the yellow and white feather groups was performed using the package DEGseq, and DEGs were screened using a significance criterion of false discovery rate (FDR) ≤ 0.05 and |log_2_(fold change)| ≥ 1 between the two groups^73^.

To further elucidate the functional roles of DEGs, GO and KEGG pathway enrichment analysis were performed using the DAVID database (https://david.abcc.ncifcrf.gov/). GO enrichment analysis can reveal the biological functions of DEGs, and KEGG is a database resource containing a collection of manually drawn pathway maps. GO terms that were significantly annotated (*P* < 0.05) were considered significantly enriched.

### Real-time quantitative reverse transcription PCR (qRT-PCR) verification of RNA-seq data

Four genes (*MFSD12*, *TYR*, *NR1H4* and *THRSP*) were selected to measure expression levels in feather follicles of 7- and 11-week old chickens, in order to verify the RNA-seq data using qRT-PCR. Five key pigment genes (*TYRP1*, *DCT*, *PMEL*, *MLANA* and *HPGDS*) were specifically chosen to assess their function in melanogenesis; to this end, we measured their expression levels in all samples of 3-, 5-, 7-, and 11-week old chickens. The primers for these genes are described in Table S5.

The qRT-PCR was performed on a CFX96TM Real-Time System (Bio Rad, Hercules, CA, USA) using Real Master Mix SYBR Green I (Tiangen). *GAPDH* was used as a housekeeping gene in all qRT-PCR experiments. Five biological replicates per group and three technical replicates per sample were used for qRT-PCR. The 2^−ΔΔCT^ method was used to calculate gene expression levels^74^. Significant differences between the two groups were evaluated using unpaired Student’s *t* tests. All results are shown as the means ± standard error (SE). *P* < 0.05 was considered significant and *P* < 0.01 extremely significant.

## Supplementary information

Supplementary Information 1.

Supplementary Information 2.

Supplementary Information 3.

Supplementary Information 4.
