# Supplementary material for: Transcriptome analysis of feather follicles reveals candidate genes and pathways associated with pheomelanin pigmentation in chickens
Source: Sci Rep. 2020 Jul 21;10:12088. doi: 10.1038/s41598-020-68931-1 (PMC7374586; doi:10.1038/s41598-020-68931-1)
Supplement: Supplementary file 1 — Supplementary Information 1. [file 41598_2020_68931_MOESM1_ESM.docx]

**Transcriptome analysis of feather follicles reveals candidate genes and pathways associated with pheomelanin pigmentation in chickens**

Xiaotong Zheng, Bo Zhang, Yawen Zhang, Haian Zhong, Ruixue Nie, Junying Li, Hao Zhang*, Changxin Wu

National Engineering Laboratory for Animal Breeding, College of Animal Science and Technology, China Agricultural University, Beijing, 100193, China

* Corresponding author: zhanghao827@163.com.

**Supplementary information**

[Supplementary Figures: 3](#_Toc19154)

[Supplementary Figure S1 3](#_Toc19437)

[Supplementary Figure S2 4](#_Toc29864)

[Supplementary Figure S3 5](#_Toc9797)

[Supplementary Figure S4 6](#_Toc15923)

[Supplementary Figure S5 7](#_Toc8072)

[Supplementary Tables: 9](#_Toc12760)

[Supplementary Table S1 9](#_Toc752)

[Supplementary Table S2 10](#_Toc14021)

[Supplementary Table S3: Please see the Excel file 11](#_Toc10331)

[Supplementary Table S4: Please see the Excel file 11](#_Toc25863)

[Supplementary Table S5: Please see the Excel file 11](#_Toc4269)

# Supplementary Figures:

## **Supplementary Figure S1**


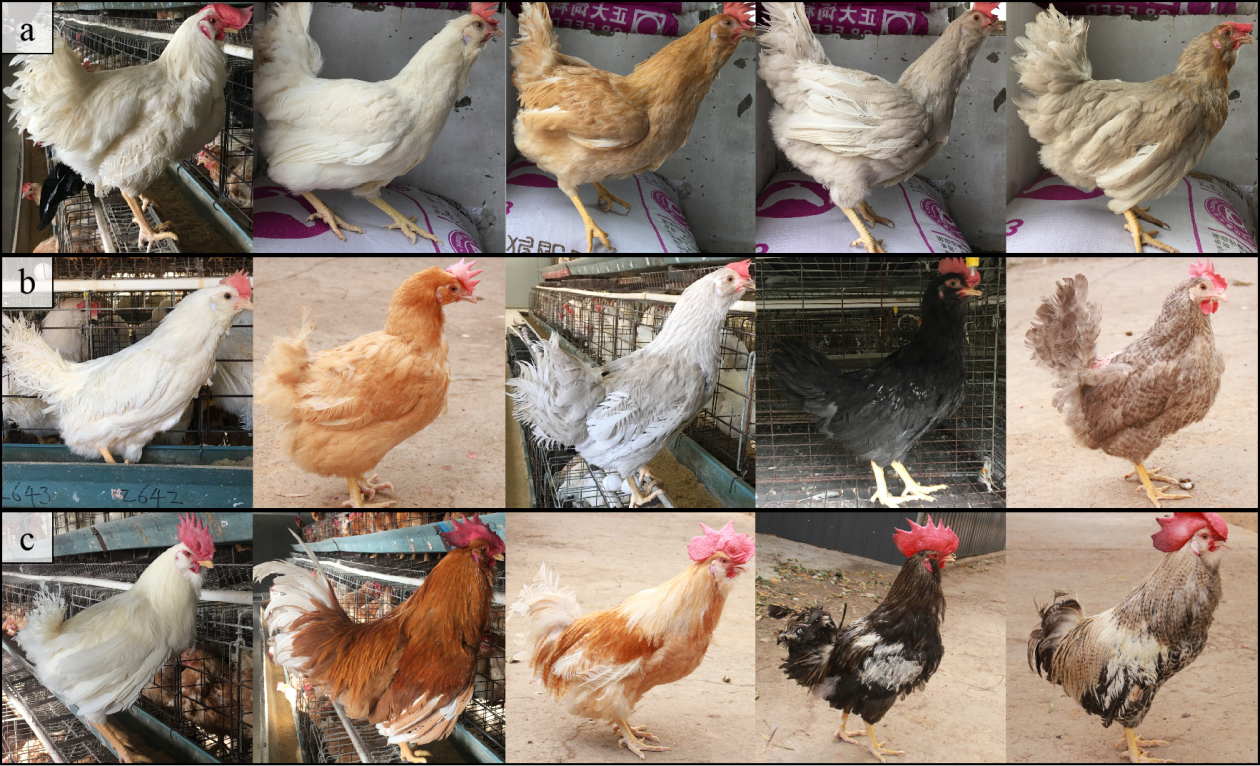


**Figure S1.** Photos of Huiyang Bearded chicken (HB) and White Leghorn chicken (WL) hybrid population. (**a**) Cocks and hens of F1 hybrid population. (**b**) Hens of F2 hybrid population. (**c**) Cocks of F2 hybrid population. In F2 roosters, there are also individuals with grey plumage, whose color is similar to that of F2 grey-feathered hens. We replaced what should be F2 grey-feathered cocks with yellow and white barred pattern plumage ones.

## **Supplementary Figure** S2


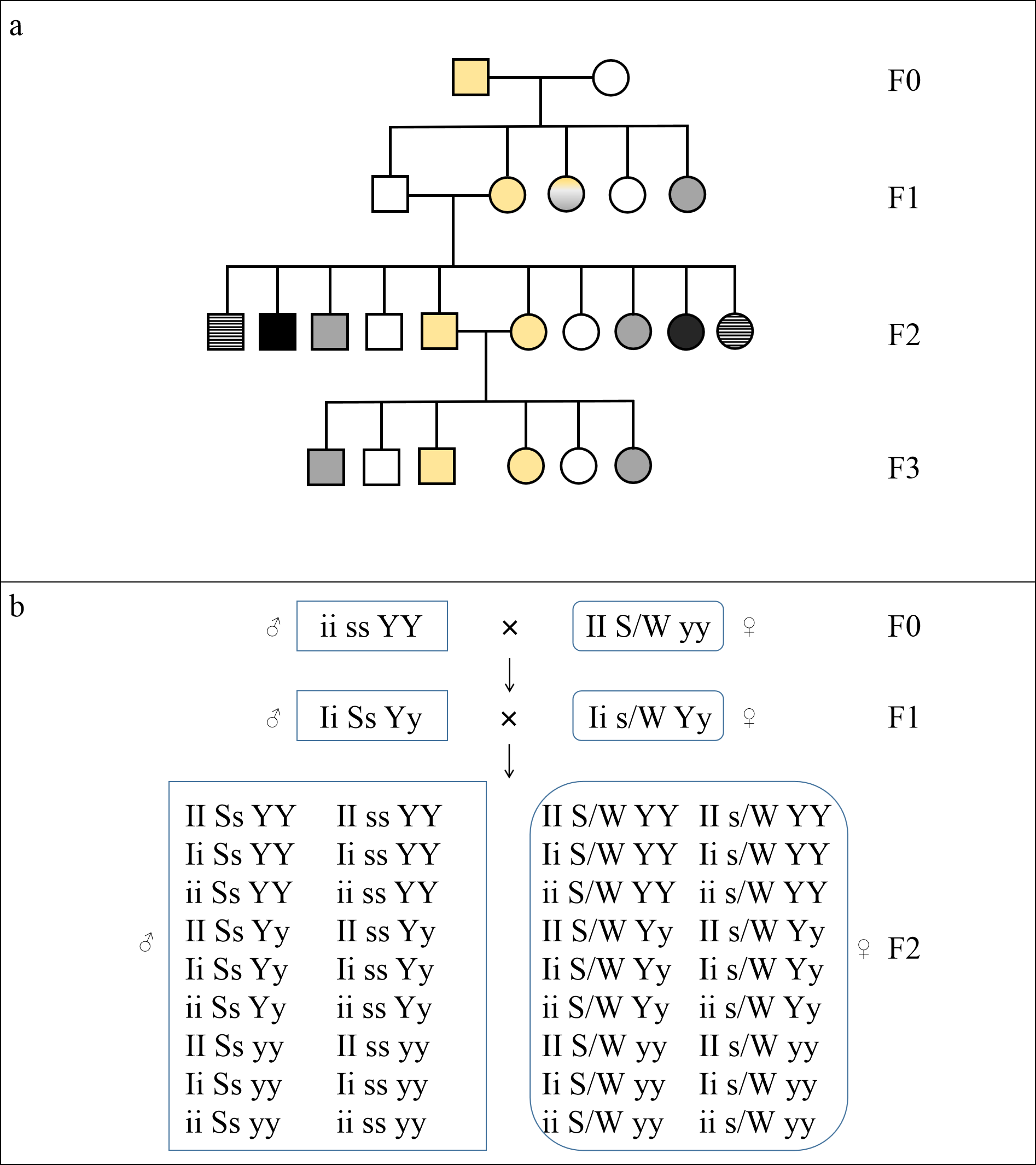


**Figure S2.** Schematic diagram of the plumage colours and genotypes of chickens in HB and WL hybrid populations (**a**) Schematic diagram of plumage colours of F0, F1, F2 and F3 populations. (**b**) Genotypes of chickens in F0, F1 and F2 populations. Yellow plumage trait is considered to be dominant inheritance controlled by single gene, represented as Y. I represents the *Dominant white* allele and S represents *Silver* allele.

## **Supplementary Figure** S3


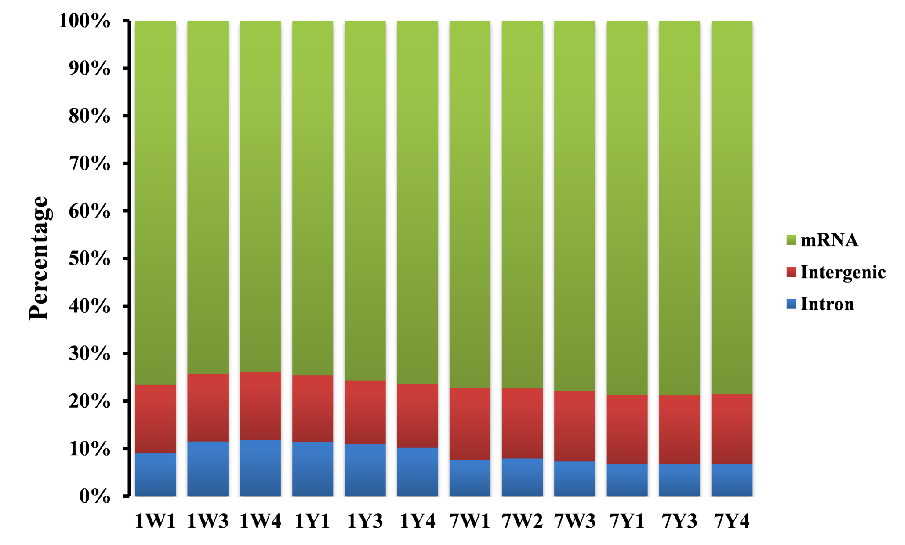


**Figure S3.** Statistical diagram for the distribution of reads mapped to the reference genes.

## **Supplementary Figure** S4

**
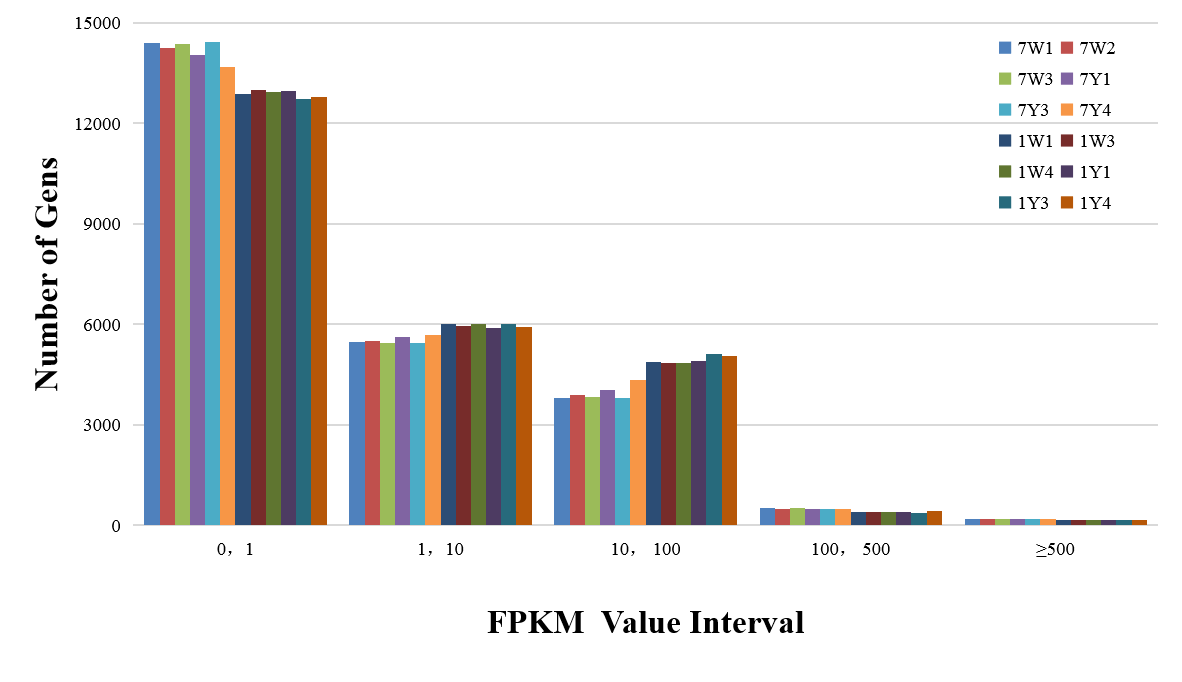
**

**Figure S4.** Histogram of fragments per kilobase of exon per million mapped fragments (FPKM) distributions in each sample.

## **Supplementary Figure** S5

**
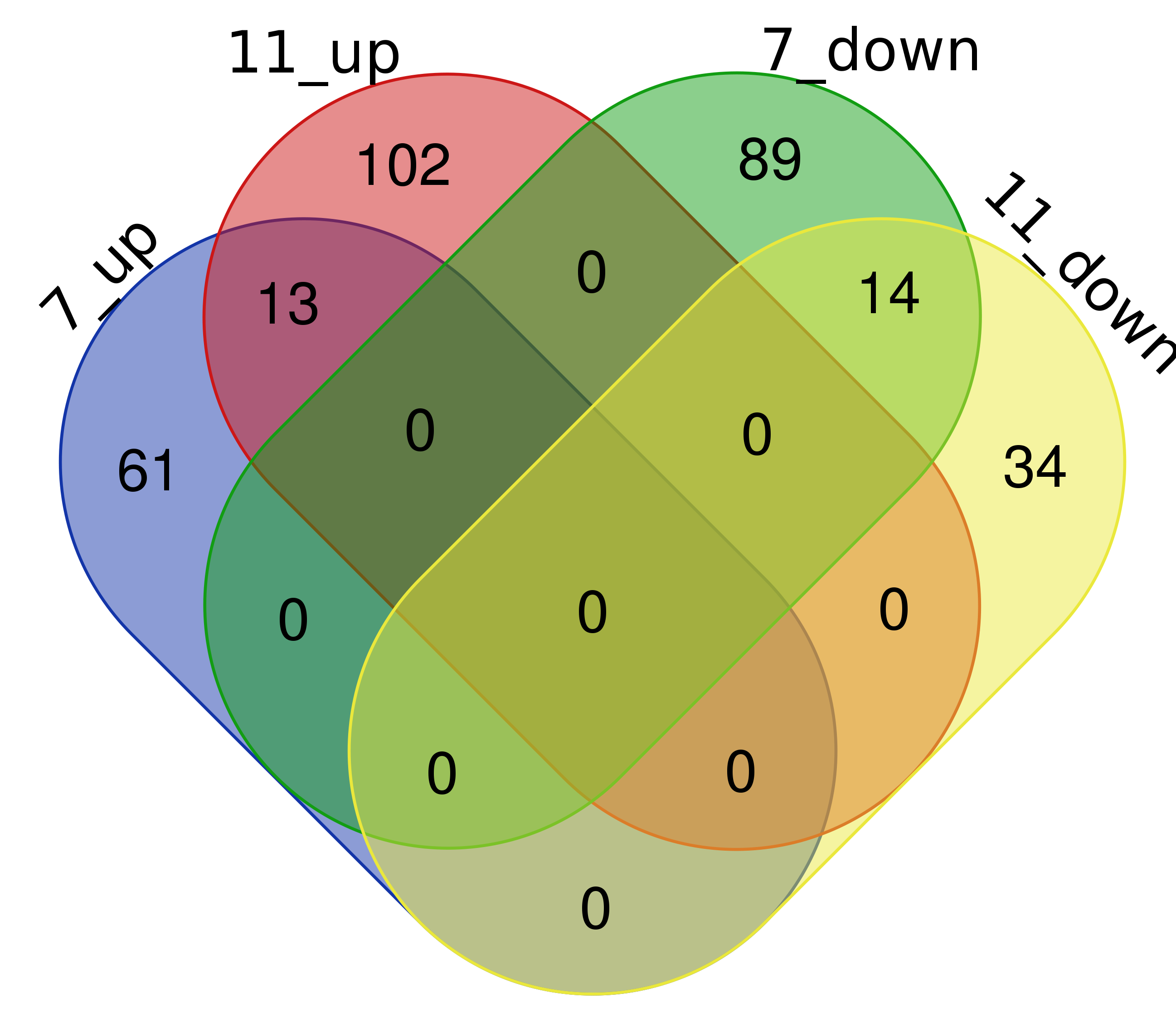
**

**Figure S5.** Counts of overlapping differentially expressed genes (DEGs) between 7- and 11-week ages.

**Supplementary Tables:**

**Supplementary Table S1**

| Generation | Plumage colour | Number of cocks | Number of hens |
| --- | --- | --- | --- |
| F1 | White | 684 | 34 |
|  | Yellow | 0 | 305 |
|  | Grey | 0 | 37 |
|  | Grey plume yellow head | 0 | 309 |
| F2 | White | 68 | 72 |
|  | Yellow | 49 | 31 |
|  | Grey | 13 | 21 |
|  | Black | 2 | 1 |
|  | Black and white barred | 17 | 12 |
| F3 | White | 72 | 45 |
|  | Yellow | 79 | 104 |
|  | Grey | 9 | 7 |

**Table S1.** Statistics on the number of different plumage colour chicken in HB and WL hybrid populations.

**Supplementary Table S2**

| Groups | No. of sample | Dominant white locus | recessive white locus | Silver locus |
| --- | --- | --- | --- | --- |
| White plumage chicken at 7-week age | 7W1 | II | CC | s/W |
|  | 7W2 | Ii | CC | s/W |
|  | 7W3 | II | CC | s/W |
| Yellow plumage chicken at 7-week age | 7Y1 | Ii | CC | s/W |
|  | 7Y3 | Ii | CC | s/W |
|  | 7Y4 | II | CC | s/W |
| White plumage chicken at 11-week age | 11W1 | II | CC | s/W |
|  | 11W3 | II | CC | s/W |
|  | 11W4 | II | CC | s/W |
| Yellow plumage chicken at 11-week age | 11Y1 | Ii | CC | s/W |
|  | 11Y3 | Ii | CC | s/W |
|  | 11Y4 | II | CC | s/W |

**Table S2.** The pigment genotype identification results of samples used for RNA-seq.

II represents homozygote of the *Dominant white* locus, Ii represents heterozygote of the *Dominant white* locus, CC represents sample without *recessive white* locus, s/W represents sample without *Silver* locus.

**Supplementary Table S3: Please see the Excel file**

Table S3. RNA-seq quality, read counts and mapping rates of 12 libraries.

**Supplementary** **Table S****4: Please see the Excel file**

Table S4: All differentially expressed genes (DEGs) and fragments per kilobase million mapped reads (FPKM) of per sample.

**Supplementary** **Table S5: Please see the Excel file**

Table S5: Primers used for quantitative RT-PCR.
